# Ocular and Systemic Phenotyping of Bardet–Biedel Syndrome Type 7 (BBS7) in a Palestinian Male: Case Report and Literature Review

**DOI:** 10.1155/crop/7604431

**Published:** 2026-07-01

**Authors:** Ibrahim Taha, Lubna Abusamra, Sundus F. Shalabi, Khalil Huraibat, Liana Al-Labadi, Khaled R. Beshtawi, Feras Abujaber, Mutasem Khalaf, Rema Obar

**Affiliations:** ^1^ Optometry Department, Arab American University, Ramallah, State of Palestine, aauj.edu; ^2^ Faculty of Medicine, Arab American University, Ramallah, State of Palestine, aauj.edu; ^3^ Department of Oral and Maxillofacial Surgery, Oral Medicine and Periodontology, Faculty of Dentistry, University of Petra, Amman, Jordan, uop.edu.jo; ^4^ Department of Industrial Chemistry, Faculty of Sciences, Arab American University (AAUP), Jenin, State of Palestine, aauj.edu; ^5^ Department of Audiolgy and Speech Therapy, Birzeit University, Birzeit, State of Palestine, birzeit.edu

## Abstract

**Introduction:**

Bardet–Biedl syndrome (BBS) is a rare autosomal recessive ciliopathy with at least 26 identified causative genes. BBS7 accounts for approximately 1.5% of cases. The syndrome is characterized by retinal degeneration, obesity, polydactyly, intellectual disability, and renal anomalies. BBS7 encodes a core subunit of the BBSome complex, which is essential for ciliary function and intracellular signaling.

**Case Presentation:**

We report a 9‐year‐old Palestinian boy with BBS7, confirmed by whole exome sequencing identifying a homozygous pathogenic variant in the BBS7 gene [c.712_715del]; both parents were heterozygous carriers. To our knowledge, this is the first genetically confirmed case of BBS7 in Palestine. Written informed consent was obtained from the father, the legal guardian. The patient exhibited postaxial polydactyly, generalized obesity (BMI 32.8), developmental delay, learning difficulties, hypothyroidism, and mild craniofacial dysmorphism. Ocular findings included a best‐corrected visual acuity of 20/33 bilaterally, compound myopic astigmatism (spherical equivalents: −7.12 D OD and −7.25 D OS), and 15 prism diopters of constant left exotropia. Optical coherence tomography revealed marked thinning of the retinal nerve fiber layer and ganglion cell complex consistent with progressive retinal degeneration. Fundus imaging demonstrated tilted optic discs, bone spicule pigmentation, and peripapillary atrophy. Full‐field electroretinography confirmed rod‐cone dystrophy with significantly reduced amplitudes and prolonged implicit times. Corneal topography showed no evidence of keratoconus.

**Discussion:**

This case contributes novel region‐specific clinical and genetic data on BBS7, with comprehensive ophthalmic phenotyping including OCT, full‐field ERG, and corneal topography. It underscores the value of early multidisciplinary evaluation and genetic testing, particularly in consanguineous populations. Continued reporting of genetically confirmed BBS7 cases will strengthen understanding of genotype–phenotype correlations and support improved clinical management.

## 1. Introduction

Bardet–Biedl Syndrome (BBS) is a rare ciliopathic dysfunction, characterized by the involvement of multiple organs and is inherited in an autosomal recessive manner, although some studies reported an oligogenic inheritance. Currently, there are at least 26 identified genes associated with BBS. Among these, the *BBS1* gene is the most commonly implicated, accounting for approximately 23% of cases, followed by *BBS10* and *BBS2*, which are responsible for around 15% and 10% of cases, respectively [1].

The systemic and ocular manifestations vary between the affected individuals as well as the type of gene involved and are categorized into primary and secondary clinical features [2]. The key ocular manifestation of BBS is retinal degeneration, either rod‐cone or cone‐rode dystrophies [3]. Retinal degeneration results in impaired vision and visual field defects, in addition to night vision abnormalities. Clinically, the diagnosis of retinal degeneration is confirmed using an electroretinogram (ERG), which shows nonrecordable signals or severely diminished signals in those patients. In addition, the fundus appearance shows features of retinitis pigmentosa. Other ocular manifestations were also reported, such as refractive errors, cataracts, strabismus, and nystagmus [4, 5].

Additional primary BBS systemic features include central obesity, renal abnormalities, postaxial polydactyly, intellectual disability, and genital system anomalies [6]. Other features such as syndactyly, brachydactyly, diabetes, anosmia, dental crowding, and facial dysmorphism are considered secondary BBS features. According to Beales et al. [7], the diagnosis of BBS requires four primary features or three primary features plus two secondary features.

The global prevalence of BBS varies widely across different populations. In Northern Europe, the estimated occurrence is approximately 1 in 160,000 [8].

Although BBS has been previously documented in Palestine, those reports involved different genetic subtypes. Karmi et al. [9] described a case with pathogenic *FBN3* variants, and Milhem et al. [10] reported a case with a *MKKS* mutation. Neither included detailed ophthalmic evaluation [9, 10]. In contrast, our case is the first genetically confirmed instance of BBS7 in the region and uniquely features comprehensive ocular phenotyping—including corneal topography, OCT, and full‐field ERG.

In the scope of this investigation, we report the systemic and ocular phenotypes associated with BBS7 in a juvenile Palestinian child.

## 2. Case Presentation

A 9‐year‐old Palestinian male presented to the optometry clinic at the Arab American University of Palestine for assessment related to suspected retinitis pigmentosa. His birth history was significant for a cesarean section delivery due to maternal indications, with a recorded birth weight of 2.7 kg. Physical examination at birth identified postaxial polydactyly. The patient′s family history was significant for consanguinity, as his parents are first cousins. Both parents are healthy, possess a good level of educational attainment, and have no reported genetic disorders. They have four additional children (three sons and one daughter), all of whom are unaffected. The mother reported one prior miscarriage, but there were no additional familial indications of hereditary diseases.

The patient′s developmental history included mild learning difficulties and poor academic performance. Ocular history was positive for refractive error and retinal degeneration. A comprehensive clinical evaluation revealed involvement of multiple systems, including postaxial polydactyly of the hands and feet, generalized obesity, and mild craniofacial dysmorphism characterized by a depressed nasal bridge, deep‐set eyes, and retrognathia.

Laboratory findings indicated hypothyroidism, with the free thyroxine (FT4) level measured at 0.86 ng/dL and thyroid‐stimulating hormone (TSH) level at 8.68 *μ*IU/mL. A recent physical assessment documented a body weight of 52 kg (exceeding the normal reference range of 22–38 kg for age) and a height of 126 cm (within the normal range of 121–144 cm). The body mass index (BMI) calculated is 32.8 (corresponding to the > 99th percentile for age and sex, consistent with severe obesity by pediatric BMI‐for‐age classification). Blood pressure readings were within normal limits. The patient did not present with any kidney abnormalities or other systemic diseases. Renal ultrasound was performed and showed no structural abnormalities, with normal kidney size and echogenicity bilaterally. Genitourinary examination did not reveal any clinically significant anomalies. Developmental and cognitive assessment confirmed mild intellectual disability and learning difficulties, consistent with a primary BBS7 feature. The endocrine evaluation was limited to thyroid function in the context of this clinical presentation; further metabolic and endocrinological workup, including fasting glucose, lipid profile, and assessment for hypogonadism, is recommended as part of ongoing multidisciplinary care. These normal and documented systemic findings are reported comprehensively in accordance with CARE guidelines for syndromic case evaluation.

### 2.1. Participant Consent Statement

Written informed consent was obtained from the patient′s father, who is the legal guardian of the child, prior to any study procedures after being briefed on the study′s purpose, methods, and potential risks. The patient′s family was assured that they could withdraw from the study at any time without affecting the patient′s ongoing clinical care. The consent process complied with the Declaration of Helsinki and applicable local regulations. This case report was conducted in accordance with CARE (CAse REport) guidelines.

## 3. Materials and Methods

### 3.1. Genetic Analysis

DNA was extracted from the blood of the patient and his parents using a GenElute Blood Genomic kit (Sigma, St. Louis, Missouri, United States) per the manufacturer′s manual. Whole exome sequencing (WES) was conducted in a trio format using the HiSeq 1000 platform (Illumina, 2 × 100 bp), which allows for the comprehensive analysis of all protein‐coding regions of the genome to identify potential pathogenic variants. The data generated from the WES analysis have not been deposited in a public repository but are available upon reasonable request. Identified variants in the patient and parents′ genomes were confirmed by Sanger sequencing. Primers were designed via Primer3Plus (https://www.primer3plus.com/30August2023); and PCR was done using the PrimeSTAR GXL DNA kit. PCR products were cleaned with the A′SAP‐ArticZymes method, then sequenced with BigDye Terminator V3.1 Cycle Sequencing Kit on a 3500 XL Series Genetic Analyzer.

### 3.2. Bioinformatic Analysis

#### 3.2.1. The Human Phenotype Ontology (HPO) Terms

Based on the clinical assessment of the patient and his physicians′ reports, the HPO database was utilized to identify HPO terms that describe the patient′s phenotypes and clinical picture (https://hpo.jax.org/app/30 August 2022) (Table 1).

**Table 1:** HPO terms describe the patients′ phenotypes.

HPO term	HPO code
Polydactyly	HP:0010442
Obesity	HP:0001513
Intellectual disability, mild	HP:0001256
Learning disability	HP:0001328
Depressed nasal bridge	HP:0005280
Deeply set eye	HP:0000490
Retrognathia	HP:0000278
Hypothyroidism	HP:0000821
Myopia	HP:0000545
Astigmatism	HP:0000483
Chorioretinal degeneration	HP:0200065
Rod‐cone dystrophy	HP:0000510
Abnormal electroretinogram	HP:0000512
Exotropia	HP:0000577
Brachydactyly	HP:0001156

#### 3.2.2. Filtering and Variant Prioritization

VCF files were created using the ISAAC pipeline [11]. ISAAC aligner and ISAAC variant caller were used for data alignment and variant calling. The mean read depth (coverage) achieved was 65×, with over 91% of target exome bases covered at ≥ 20× depth, ensuring adequate sensitivity for variant detection. For variant annotation and interpretation, enGenome eVai platform (http://evai.engenome.com) was used, which relies on artificial intelligence with ACMG, AMP, and ClinGen guidelines. Using eVai ensures accurate reporting and classification of genomic variants. Variants were assessed for effects, filtered by phenotypes (HPO), and assigned pathogenicity scores.

Using eVai, two filters were applied:

##### 3.2.2.1. Filter 1

High‐quality variants (QUAL ≥ 30) with a pathogenicity score ≥ 5 were selected for the patient.

##### 3.2.2.2. Filter 2

Variants (QUAL ≥ 30) associated with each HPO term in Table 1 were selected. Variants with low impact (Pathogenicity score < 0) and deep intronic variants were excluded.

#### 3.2.3. Variant Interpretation

Variants from Filters 1 and 2 were examined based on their position, gnomAD frequency, mutation effect, inheritance mode, and data from mutation databases (ClinVar, LOVD, HGMD), as well as insights from literature, related diseases, and phenotypes using MalaCard, OMIM, and HPO. Furthermore, computational prediction tools such as eVai pathogenicity score, ACMG, SIFT, MVP, FATHMM, MutationTaster, M‐CAP, and CADD were assessed.

#### 3.2.4. Ocular Assessment

A comprehensive ophthalmological assessment of the patient was done, assessing visual acuity, refraction, and ocular alignment (using a cover test). External ocular health was examined using an SL9800 CSO slit lamp.

Additionally, the integrity of the posterior segment was thoroughly assessed via fundus examination, performed with a Topcon TRC‐NW8 nonmydriatic camera.

To obtain cross‐sectional images of the retina, Optovue iVue optical coherence tomography (OCT) was employed. For a comprehensive analysis of the corneal status, the OCULUS Pentacam was utilized.

The full‐field electroretinogram (ffERG) was conducted using the Metrovision MonPackONE system, following the International Society for Clinical Electrophysiology of Vision (ISCEV) standards [12]. Corneal electrodes were used for signal acquisition, with reference electrodes at the outer canthi and a ground electrode on the forehead. A bandpass filter of 0.3–300 Hz captured the a‐wave (photoreceptor activity) and b‐wave (inner retinal activity).

For scotopic testing, the patient underwent 20 min of dark adaptation, followed by Scotopic 0.01 (rod‐specific) and Scotopic 10.0 (mixed rod‐cone) recordings. After 10 min of light adaptation, Photopic 3.0 assessed cone responses, and Photopic 3.0 Flicker (30 Hz) tested cone temporal function.

Stimuli were delivered using a Ganzfeld dome, with calibration performed prior to testing to ensure accuracy. Signal quality was monitored throughout, with repeated measurements taken as needed [13]. This protocol provided a comprehensive assessment of retinal function.

#### 3.2.5. Ethical Considerations

A written informed consent was obtained from the father, who is the legal guardian of the child, after being briefed on the study′s purpose, methods, and risks. The patient and their family were assured that they could withdraw from the study at any time without affecting their ongoing care.

### 3.3. Statistical Methods and Reporting

This study is a single‐patient case report; therefore, formal inferential statistical analyses were not applicable or performed. All clinical measurements are reported descriptively, including quantitative electroretinography values, OCT measurements, and biometric data, presented with their exact values as obtained. Normative reference values cited are those defined in the relevant published ISCEV standards and peer‐reviewed publications. No a priori significance levels or hypothesis testing were employed. This report follows the SAMPL (Statistical Analyses and Methods in the Published Literature) guidelines for descriptive reporting, and adheres fully to the CARE guidelines for case report standardization.

## 4. Results

### 4.1. WES and Bioinformatic Results

Using the eVai software for variant analysis and filtering, significant genetic variants were identified based on the criteria established by Filters 1 and 2, as follows:

#### 4.1.1. Filter 1

In the *BBS7* gene, a homozygous variant c.712_715del was identified, with an eVai pathogenicity score of 7, suggesting its potential significance in BBS Type 7 (OMIM:607590). Additionally, a heterozygous variant c.2177 T > C was identified in the *MEFV* gene, which is associated with familial Mediterranean fever (FMF) (OMIM: 608107), and was assigned an eVai pathogenicity score of 5.

#### 4.1.2. Filter 2

the variant c.712_715del in the *BBS7* gene was only found, which is related to the HPO terms that describe the patient′s phenotype.

Each variant was independently assessed using the in silico predictive tools outlined in Section 3.2.3, “Variant Interpretation,” under Section 3, “Materials and Methods”.

The *BBS7* gene variant NM_176824.4:c.712_715del (p.Glu238Serfs∗7), a homozygous frameshift deletion resulting in a premature termination codon, has been classified as pathogenic based on the criteria of the ACMG guidelines [14], meeting the standards of PM2, PVS1, and PP5. Specifically: PVS1 was applied because the variant causes a frameshift leading to a premature stop codon (p.Glu238Serfs∗7), predicted to result in loss of function of the BBS7 protein; PM2 was applied because the variant is absent or present at an extremely low frequency in population databases (MAF = 0.00005181 in gnomAD); and PP5 was applied because the variant has been previously classified as pathogenic in ClinVar by independent submitters. The patient is confirmed to carry this variant in the homozygous state, consistent with the autosomal recessive inheritance pattern of BBS7. Furthermore, the ClinVar database has also recognized this variant as pathogenic [15]. Supporting this classification, the computational analysis tool MutationTaster (https://www.mutationtaster.org) has described the variant as “disease‐causing” [16]. The variant is classified as a rare one, according to the gnomAD database (https://gnomad.broadinstitute.org), with a minor allele frequency (MAF) of 0.00005181 [17]. OMIM, MalaCard, and HPO databases linked this gene variant to be related to BBS7 syndrome. Using PubMed, the variant c.712_715del in *BBS7* has been previously reported [6, 16]. The presence of the mutation was validated through Sanger sequencing, and both parents have been identified as heterozygous carriers.

In our genomic analysis, a heterozygous *MEFV* c.2177 T > C (p.Val726Ala) variant was identified as an incidental finding. A notable discrepancy was observed between ACMG criteria, which designate this variant as “likely pathogenic,” and multiple in silico computational tools including SIFT, CADD, DANN, FATHMM, and MutationTaster, which unanimously classified it as “benign.” Given that the patient is only a heterozygous carrier and exhibits no clinical manifestations of FMF (an autosomal recessive disorder), this variant is reported as an incidental carrier finding of uncertain clinical significance in the context of this case.

### 4.2. Ocular Involvement

The patient′s ocular characteristics are described as follows:

#### 4.2.1. Refractive and Ocular Alignment Assessment

The patient′s visual acuity assessment demonstrated a best‐corrected visual acuity (BCVA) of 20/33 in both eyes. The cycloplegic refraction findings indicated significant compound myopic astigmatism, measured as OD: −5.00 −4.25 × 008 and OS: −5.00 −4.50 × 177.

The binocular vision assessment using a cover test revealed that the patient has constant left 15 PD exotropia (XT).

#### 4.2.2. Corneal Topography

To assess whether the patient′s high degree of astigmatism was due to corneal abnormalities, Pentacam imaging was performed. The examination showed a normal asymmetrical bowtie with superior steepening topographical patterns in both eyes. No signs of keratoconus or other corneal disorders were found based onBelin‐ABCD criteria [18]. It should be noted that quantitative indices specifically recommended for ectasia exclusion, including maximum keratometry (Kmax), thinnest pachymetry, and the Belin–Amórous deviation (BAD‐D) score, were not available from the clinical report at the time of this case evaluation. Their absence represents a limitation of the current corneal assessment, and these parameters are recommended for documentation in future follow‐up examinations. Regarding OCT measurements, normative comparisons were performed using published pediatric reference values [19], as the Optovue iVue device‐specific pediatric normative database was not available for this patient′s age group; all comparisons should therefore be interpreted with appropriate caution.

#### 4.2.3. OCT

**Figure 1:**
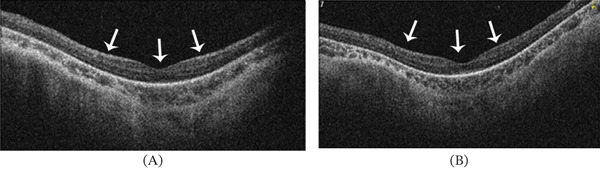
Cross‐sectional OCT of the (A) left eye and (B) right eye showing a disruption and thinning of the outer retinal layers (arrows), mainly the photoreceptor layer which is related to the retinal degeneration feature of BBS7, in addition to RPE changes. The images also show that the thinning is more pronounced in the right eye.

In terms of OCT analysis, the retinal nerve fiber layer (RNFL) and ganglion cell complex (GCC) measurements revealed marked reductions, consistent with progressive retinal degeneration (Figure 1). These details align with the observed clinical picture and are indicative of the characteristic ocular pathology associated with BBS [20].

The measurements of the RNFL and GCC, as detailed in Table 2, revealed markedly reduced values. These findings are indicative of progressive retinal degeneration in the patient, correlating with the expected pathophysiological changes associated with BBS7.

**Table 2:** Measurements of the retinal nerve fiber layer (RNFL) and ganglion cell complex (GCC).

Analysis	OD	OS
Average GCC (*μ*m)	47	57
Superior GCC (*μ*m)	42	53
Inferior GCC (*μ*m)	52	61
S‐I (*μ*m)	–10	–8
FLV %	31.36	35.30
GLV %	47.85	42.11
Average RNFL (*μ*m)	68	72
Superior RNFL (*μ*m)	71	89
Inferior RNFL (*μ*m)	65	56
S‐I (*μ*m)	6	33

#### 4.2.4. Fundus Photography

**Figure 2:**
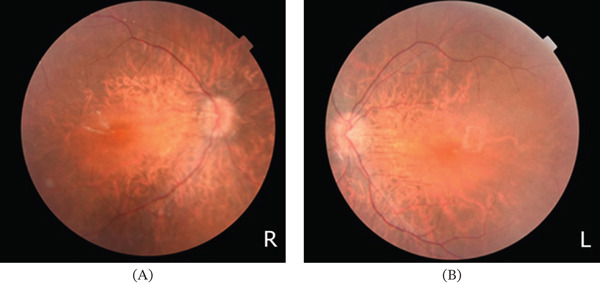
Fundus photography of the (A) right eye and (B) left eye demonstrating tilted optic discs, bone spicule pigmentation, peripapillary atrophy, and visible underlying choroidal vasculature indicative of retinal thinning.

Fundus imaging of the right eye (A, left) and left eye (B, right) showed tilted optic discs, bone spicule pigmentation, peripapillary atrophy and visible underlying choroidal vasculature indicative of retinal thinning (Figure 2).

#### 4.2.5. Electrophysiology ERG

A ffERG was performed using the Metrovision MonPackONE system, in accordance with the standards of the ISCEV, 2022. The patient underwent both scotopic and photopic testing after appropriate dark and light adaptation periods. Corneal electrodes were used, and all stimuli were presented through a calibrated Ganzfeld dome.

The ERG results revealed a marked reduction in both a‐wave and b‐wave amplitudes across all test conditions, with prolonged implicit times—particularly under scotopic conditions. These findings are consistent with rod‐cone dystrophy, as typically observed in BBS. The responses remained recordable, although severely diminished, especially in the rod pathway.

Quantitative data from the ERG testing are presented in Table 3. The patient′s values are compared with normal ISCEV reference values, highlighting the significant decline in retinal function.

**Table 3:** Full‐field ERG responses in the patient compared with ISCEV normative values.

ERG test	Eye	a‐wave amplitude (*μ*V)	a‐wave implicit iime (ms)	b‐wave amplitude (*μ*V)	b‐wave implicit time (ms)	Normal b‐wave amplitude (*μ*V)	Normal implicit time (ms)
Scotopic 0.01 (rod)	OD	6	85	18	130	> 100	~90
	OS	7	83	22	125		
Scotopic 10.0 (mixed)	OD	55	23	75	100	> 150	~80
	OS	60	21	82	98		
Photopic 3.0 (cone)	OD	22	16	48	34	> 75	~30
	OS	25	17	52	32		
Photopic 3.0 Flicker (30 Hz)	OD	—	—	40	30	> 60	~28
	OS	—	—	45	28		

These results further support the diagnosis of rod‐cone dystrophy, a hallmark ocular manifestation of BBS, with greater dysfunction noted in the rod system. Although still recordable, the amplitudes are significantly below the normal range, and the implicit times are delayed, indicating widespread retinal degeneration. The term “recordable” in this context denotes that signal amplitudes exceeded the noise floor of the recording system, although they were severely diminished. Normal reference ranges presented in Table 3 are derived from published ISCEV normative data [13], which represent population‐level reference intervals for adult subjects; age‐specific pediatric normative data for this device were not available. The ERG pattern is consistent with a predominantly rod‐cone dystrophy phenotype, in which rod‐pathway dysfunction is disproportionately more severe than cone dysfunction. Contextualizing the disease stage: in a 9‐year‐old child, the presence of markedly reduced but still recordable ERG responses suggests moderately advanced retinal degeneration, consistent with the natural history of BBS7 retinopathy at this age. Visual field testing was not performed due to age‐related limitations in patient cooperation; this is acknowledged as a limitation of the current evaluation, and formal perimetric assessment is recommended at the next follow‐up visit.

## 5. Discussion

We present a case of a 9‐year‐old Palestinian boy diagnosed with BBS7. He exhibited key features of the syndrome, including retinal degeneration, postaxial polydactyly, obesity, and a consanguineous family background. Although BBS7 accounts for only about 1.5% of BBS cases, its identification is important for understanding the disorder and guiding genetic counseling. The prevalence of BBS7 in Palestine remains unknown, highlighting a gap in local genetic data.

BBS is classified as a ciliopathy. Ciliopathies are inherited disorders characterized by primary cilium dysfunction. Primary cilia are organelles made of microtubules protruding from the apical surfaces of most cells [21]. Primary cilia are important in many signaling pathways such as hedgehog (Hh), transforming growth factor beta (TGF‐beta), and WNT signaling that are essential in regulating cell polarity, differentiation, and migration of cells during embryonic development and in maintaining tissue homeostasis [22]. Essential to the function, composition, and formation of primary cilia is the BBsome. The BBSome is a complex composed of eight BBS proteins (BBS1, BBS2, BBS4, BBS5, BBS7, BBS8, BBS9 and BBIP10/BBS18) that aids in transporting signaling molecules through the cilia [21].

To date, over 20 genes have been implicated in the BBS. The *BBS7* gene was initially identified in 2003 as a novel gene associated with BBS, encoding a protein with structural similarities to those encoded by BBS1 and BBS2 [8]. Further studies in 2013 clarified BBS7′s role as an integral component of the BBSome structure, and it was observed that the clinical manifestations associated with *BBS7* mutations are comparable to those of other BBS genotypes. Additionally, BBS7, alongside BBS2, play a pivotal role in maintaining protein stability [23], [24].

BBS shows wide clinical variability across families due to its genetic heterogeneity and unclear genotype–phenotype correlations. A meta‐analysis of 899 patients using a “syndromic score” found that BBSome genes—particularly *BBS7*, *BBS2*, and *BBS9*—were linked to higher disease severity, especially regarding renal involvement [25].

OCT, Pentacam, ERG, fundus imaging, refraction, and binocular vision assessments provided a comprehensive evaluation of the patient′s ocular findings. Due to limited pediatric OCT norms, we compared RNFL values to those from Banc et al. [19], which revealed significantly reduced thickness (< 100 *μ*m). As GCC norms were not included in that study, we referred to other recent publications confirming typical GCC values also exceed 100 *μ*m [25].

In contrast, our patient′s GCC measurements were significantly lower, between 50 and 60 *μ*m. Notably, the comparative studies referenced herein utilized spectral‐domain OCT (SD‐OCT) technology.

Pentacam imaging was performed due to the patient′s high refractive error (OD: −5.00 −4.25 × 008, OS: −5.00 −4.50 × 177). The corneal topography showed normal sagittal curvature, elevation, pachymetry, and regularity indices, with no signs of keratoconus based on diagnostic criteria used in the Palestinian clinical setting [26].

However, visual electrophysiology in the majority of BBS cases is typically nonrecordable or markedly impaired [27]. In this case, although the patient′s ERG was significantly compromised, it remained recordable. Moreover, the GCC measurements in the left eye were somewhat better than those in the right eye, which could correlate with the relatively improved ERG outcomes for the left eye.

The literature on the BBS7 c.712_715del variant is limited but generally supports findings consistent with our case. Bin et al. [28] described an 11‐year‐old Peruvian girl with compound heterozygous BBS7 mutations (c.712_715delAGAG and c.1121C>G), presenting with obesity, polydactyly, developmental delay, mild cognitive impairment, and renal and hepatic anomalies. Ocular findings included reduced visual acuity, cone‐rod dystrophy, retinal thinning on OCT, and preserved visual fields. Unlike our case, nystagmus and cataracts were absent. The milder phenotype was attributed to the possible hypomorphic nature of the compound mutations.

In another study, Ece Solmaz et al. [16] described nine new mutations across 17 BBS genes. Among their findings was a 19‐year‐old patient with the same c.712_715del variant as our subject, showing mild intellectual disability, developmental delay, genital anomalies, polydactyly, brachydactyly/syndactyly, obesity, rod‐cone dystrophy, dental crowding, a high arched palate, and speech delay. Notably, this patient did not present with kidney or liver involvement.

Fadi et al. described 61 BBS patients, including one with compound heterozygous *BBS7* mutations (c.712_715delAGAG and c.1037+29T>A). This patient showed multiple systemic features—polydactyly, brachydactyly, obesity, speech and intellectual delays, and behavioral issues. Ocular findings included retinitis pigmentosa, reduced visual acuity, hyperopia (OD +3.00, OS +2.50), recordable ERG, bull′s eye maculopathy, cataracts, nystagmus, strabismus, and OCT abnormalities [29].

The previously mentioned cases share several features with our patient, including obesity, polydactyly, genital anomalies, renal findings, rod‐cone dystrophy, and mild cognitive impairment. However, some manifestations in our case were absent in others, underscoring the phenotypic variability of BBS—even among patients with similar mutations. This suggests that genotype–phenotype correlations in BBS7 may influence the degree of intellectual disability and other clinical outcomes.

The *MEFV* gene, associated with FMF, is prevalent among individuals of Mediterranean descent, including those with North African, Jewish, Arab, Armenian, Turkish, and Greek ancestries [30]. As an incidental finding of uncertain clinical significance in this context, a heterozygous *MEFV* c.2177T>C (p.Val726Ala) variant was identified in the patient. This gene encodes pyrin, vital in regulating inflammatory responses. Mutations in *MEFV*, such as the variant c.2177T>C found in our patient, lead to FMF, characterized by recurrent fever and inflammation [31].

FMF follows an autosomal recessive inheritance pattern, requiring two mutated genes for full manifestation, though symptoms can appear in carriers of a single mutated gene [32]. The discovery of *MEFV* mutations aids FMF diagnosis and has broader implications for understanding inflammatory processes, influencing the development of treatments like colchicine [33]. As this patient carries only a single heterozygous variant, he does not meet the genetic criteria for FMF diagnosis, and he exhibited no clinical manifestations of the disease. This finding is therefore reported as an incidental carrier variant of uncertain clinical relevance and requires no further action at this time. Clinical monitoring for FMF symptoms is advisable given the patient′s ethnic background and the population‐level prevalence of this variant.

In the context of BBS research, the insights from such genetic studies underscore the importance of WES in diagnosing complex genetic conditions, enhancing our approaches to their management and treatment [34], [35].

The use of WES has significantly improved the diagnosis of rare genetic diseases like BBS. It enables clinicians to explore genotype–phenotype correlations more effectively. WES has proved to be efficient in its valuable contribution to more than 25% improvement in the overall diagnostic approach [33].

Due to the rarity of *BBS7*, research and resources are extremely limited and transitional studies are necessary for the overall advancement and comprehensive understanding of the syndrome. This will lead to the ultimate formulation of strategies for improved outcomes for individuals suffering from BBS7 and other rare disorders.

## 6. Conclusion

This case represents the first genetically confirmed BBS7 diagnosis in Palestine, involving the known c.712_715del variant. Despite its prior documentation, the detailed ocular findings—including OCT, ERG, and corneal topography—offer valuable clinical insight. The case underscores the importance of early diagnosis, genetic testing, and multidisciplinary care, especially in populations with high consanguinity. Continued reporting of such cases can enhance understanding and management of BBS7.

## Funding

No funding was received for this manuscript.

## Disclosure

All authors have read and approved the final version of the manuscript. Ibrahim Taha had full access to all of the data in this study and takes complete responsibility for the integrity of the data and the accuracy of the data analysis. Artificial intelligence (AI) writing assistance tools were used solely for language editing and writing improvement of this manuscript. AI was not used in study design, data collection, data analysis, interpretation of results, or generation of any scientific content. All scientific conclusions, intellectual contributions, and final approval of the manuscript are the sole responsibility of the named authors.

## Ethics Statement

Ibrahim Taha affirms that this manuscript is an honest, accurate, and transparent account of the study being reported; that no important aspects of the study have been omitted; and that any discrepancies from the study as planned have been explained.

## Consent

All the patients allowed personal data processing and informed consent was obtained from all individual participants included in the study.

## Conflicts of Interest

The authors declare no conflicts of interest.

## Data Availability

The data that support the findings of this study are available from the corresponding author upon reasonable request.
